# The sit-to-stand test as a patient-centered functional outcome for critical care research: a pooled analysis of five international rehabilitation studies

**DOI:** 10.1186/s13054-022-04048-3

**Published:** 2022-06-13

**Authors:** Heather K. O’Grady, Lara Edbrooke, Christopher Farley, Sue Berney, Linda Denehy, Zudin Puthucheary, Michelle E. Kho, the International METRIC Critical Care Data Group, Ian Ball, Karen Burns, France Clarke, Deborah Cook, Aileen Costigan, Alison Fox-Robichaud, Ian Gordon, Kimberley Haines, Margaret Herridge, Tim Karachi, Vincent Lo, Alexandra MacDonell, Sunita Mathur, Alexander Molloy, Dale Needham, Amy Pastva, Julie Reid, Bram Rochwerg, Joleen Rose, Jill Rudkowski, Andrew Seely

**Affiliations:** 1 School of Rehabilitation Sciences, Faculty of Health Sciences, McMaster University, Hamilton, ON Canada; 2 Physiotherapy Department, The University of Melbourne, Parkville, VIC Australia; 3 Department of Health Services Research, Peter MacCallum Cancer Centre, Melbourne, VIC Australia; 4 Juravinski Hospital, Hamilton Health Sciences, Hamilton, ON Canada; 5 Physiotherapy Department, Austin Health, Heidelberg, Australia; 6 William Harvey Research Institute, Barts and The London School of Medicine & Dentistry, Queen Mary University of London, London, UK; 7 Adult Critical Care Unit, Royal London Hospital, Barts Health NHS Trust, London, UK; 8 Physiotherapy Department, St. Joseph’s Healthcare, Hamilton, ON Canada

**Keywords:** Intensive care units, Critical illness, Rehabilitation, Outcome assessment, Physical outcome measures, Exercise test, Sit-to-stand test

## Abstract

**Background:**

With ICU mortality rates decreasing, it is increasingly important to identify interventions to minimize functional impairments and improve outcomes for survivors. Simultaneously, we must identify robust patient-centered functional outcomes for our trials. Our objective was to investigate the clinimetric properties of a progression of three outcome measures, from strength to function.

**Methods:**

Adults (≥ 18 years) enrolled in five international ICU rehabilitation studies. Participants required ICU admission were mechanically ventilated and previously independent. Outcomes included two components of the Physical Function in ICU Test-scored (PFIT-s): knee extensor strength and assistance required to move from sit to stand (STS); the 30-s STS (30 s STS) test was the third outcome. We analyzed survivors at ICU and hospital discharge. We report participant demographics, baseline characteristics, and outcome data using descriptive statistics. Floor effects represented ≥ 15% of participants with minimum score and ceiling effects ≥ 15% with maximum score. We calculated the overall group difference score (hospital discharge score minus ICU discharge) for participants with paired assessments.

**Results:**

Of 451 participants, most were male (*n* = 278, 61.6%) with a median age between 60 and 66 years, a mean APACHE II score between 19 and 24, a median duration of mechanical ventilation between 4 and 8 days, ICU length of stay (LOS) between 7 and 11 days, and hospital LOS between 22 and 31 days. For knee extension, we observed a ceiling effect in 48.5% (160/330) of participants at ICU discharge and in 74.7% (115/154) at hospital discharge; the median [1st, 3rd quartile] PFIT-s difference score (*n* = 139) was 0 [0,1] (*p* < 0.05). For STS assistance, we observed a ceiling effect in 45.9% (150/327) at ICU discharge and in 77.5% (79/102) at hospital discharge; the median PFIT-s difference score (*n* = 87) was 1 [0, 2] (*p* < 0.05). For 30 s STS, we observed a floor effect in 15.0% (12/80) at ICU discharge but did not observe a floor or ceiling effect at hospital discharge. The median 30 s STS difference score (*n* = 54) was 3 [1, 6] (*p* < 0.05).

**Conclusion:**

Among three progressive outcome measures evaluated in this study, the 30 s STS test appears to have the most favorable clinimetric properties to assess function at ICU and hospital discharge in moderate to severely ill participants.

**Supplementary Information:**

The online version contains supplementary material available at 10.1186/s13054-022-04048-3.

## Background

Surviving critical illness to hospital discharge is only the beginning of the journey for patients leaving intensive care. Many patients will experience post-intensive care syndrome [1, 2], with impaired health-related quality of life for ≥ 5 years. [3] Muscle wasting is a major driver for functional disability, with rates of loss of 2–3%/day during critical illness [4]. Despite extensive research and high-quality trials into physical rehabilitation strategies, there are no consistent results demonstrating benefit for patients, despite level 1 evidence in other clinical settings [5]. One explanation could be the range of primary outcome measures chosen for rehabilitation trials.

The increasing focus on functional measures as primary outcomes for multicenter trials of physical, nutritional, and metabolic interventions within critical care has led to an increasing number of Core Outcomes Sets [6]. While such standardization is important, the clinimetric properties of these outcomes are likely to influence trial results. Single and composite measures currently used in the intensive care unit (ICU) demonstrate both floor (≥ 15% of participants with a minimum score) and ceiling effects (≥ 15% of participants with a maximum score) [7]. For example, the 6-min walk test and the Short Physical Performance Battery (SPPB) have floor effects at ICU discharge [8, 9] and the Physical Function in ICU Test-scored (PFIT-s) has ceiling effects at hospital discharge [9]. These measurement limitations could impair our ability to assess intervention effects. [9]

In patients recovering from critical illness, physical rehabilitation activities typically progress from lower extremity in-bed exercises to standing activities. Outcome measures such as knee extensor muscle strength [10], assistance required for standing [11], and standing repetitions [12] can objectively document patients’ progression. The sit-to-stand (STS) test has been extensively used across a wide spectrum of chronic diseases [13], and its properties have been examined, with healthy age- and sex-matched normal data available [14]. The widespread use and acceptability of the STS test stem from the fundamental ability to stand from sitting unaided contributes to independence of function and activities of daily living (e.g., getting out of bed or going to the toilet). The STS test maps to more complex measures including the Barthel Index, the SF-36, and the Functional Independence Measure (FIM), which has been used to measure long-term functional recovery from critical illness [15–17]. Proximal hip muscle strength and power are required for this movement, a muscle group noted to be more severely affected by ICU-acquired weakness [18]. Interventions targeting muscle mass, strength, and power of quadriceps at the hip and knee may appropriately be measured using the STS, a test which is functional, patient-centered and represents an important functional milestone across the recovery trajectory.

To date, the time-based 30-s STS (30 s STS) has been examined as a patient-centered outcome measure at ICU and hospital discharge in small patient cohorts [19, 20]. The feasibility and responsiveness of the STS as a primary outcome in ICU populations across the recovery trajectory remain unclear. Unknown factors include its clinimetric properties (e.g., quantitative measures of clinical utility) [21], and the mathematical behavior of data over time.

We therefore investigated the clinimetric properties of three progressive outcomes required for physical functional independence starting with knee extension strength, progressing to STS assistance, culminating with 30 s STS, documenting measurement characteristics of interest to clinicians, researchers, and patients. Two of these measures, knee extension and STS assistance, are components of the PFIT-s, a 4-item performance-based outcome measure. [22]

## Methods

We report this study using the Strengthening the Reporting of Observational Studies in Epidemiology statement. [23]

### Participants

Participants prospectively enrolled in five published critical care rehabilitation studies (I-SURVIVE [20], TryCYCLE [24], CYCLE Pilot RCT [19], eStimCycle [25], the EXERCISE trial [26]) from three countries contributed data. Investigators from each study form the International METRIC Critical Care Data Group (METRIC—esti**M** cycle **E**xercise cycle pilo**T** i su**R**v**I**ve try**C**ycle). Briefly, participants were adults (≥ 18 years) admitted to ICU, were ventilated, previously independent, and deemed at greatest risk of future functional disability. Full inclusion and exclusion criteria for each study are included in Additional file 1: Table 1.

In I-SURVIVE, the inter-rater reliability of the PFIT-s and 30 s STS was assessed amongst 42 participants across two Canadian ICUs (enrolled between October 2016 and December 2017) [20]. TryCYCLE assessed the safety of an early in-bed cycling protocol in a single-center Canadian prospective cohort of 33 participants (October 2013–August 2014) [24]. Sixty-six participants were enrolled across seven Canadian ICUs in the CYCLE Pilot RCT, which assessed the feasibility of early in-bed cycling plus routine physiotherapy compared to routine physiotherapy alone (May 2015–June 2016) [19]. The eStimCycle multicenter RCT enrolled 162 participants across four hospitals in Australia and the USA, evaluating the effect of functional electrical stimulation-assisted cycle ergometry on physical and cognitive outcomes (August 2014–December 2018) [25]. EXERCISE, a single-center Australian RCT, assessed the effectiveness of an intensive physiotherapy program spanning ICU admission to the outpatient setting compared to usual care among 150 participants (May 2007–August 2009) [26]. In contrast to a meta-analysis of efficacy studies where population heterogeneity limits pooling, clinical heterogeneity across our studies enhances the clinimetric evaluation of outcome measures.

### Outcome measures

We included three physical outcome measures: knee extensor strength, STS assistance, and the 30 s STS test [12, 27] (Additional file 1: Table 2). Knee extensor strength was assessed using manual muscle testing (MMT) and scored using the Medical Research Council (MRC) system. MRC scores range from 0 (no muscle contraction) to 5 (movement of muscle against gravity with full resistance) [28–30]. In each study, the MRC scoring system was used to assign a PFIT-s score ranging from 0 to 3; higher scores reflected greater strength [11, 22]. An MMT [29, 30] grades 0, 1, or 2 represented a PFIT-s score of 0; MMT grade 3 represented 1; MMT grade 4 represented 2, and MMT grade 5 represented 3. All studies recorded knee extension using the PFIT-s; however, not all studies documented individual MRC scores, and thus, we analyzed the PFIT-s. For STS assistance, a PFIT-s score of 0 represented a participant unable to perform the test; 1 represented a two-person assist; 2 represented one-person assist, and 3 represented no assist. For the 30 s STS test, participants completed as many full STS repetitions as possible in 30 s, using their arms if needed; higher scores represented greater strength and function. [12, 27]

### Procedures

In each study, acute care physiotherapists and/or physiotherapy assistants were trained and completed outcomes assessment. Additional file 1: Table 2 summarizes outcomes and time points from each study.

### Data analysis

From each study’s main dataset, we exported the following data at ICU and hospital discharge: anonymized participant identification code, knee extensor strength (PFIT-s), STS assistance (PFIT-s), 30 s STS repetitions (including whether arms were used), and reasons for missing data. If a participant did not complete an assessment because of a physical limitation or because the assessor perceived that they were unable, we scored these according to the PFIT-s (“0” (unable)). No identifying data were included in our pooled dataset.

Participants were considered “potentially eligible” for an assessment if they were enrolled in a study that assessed a given outcome at the relevant time point. Participants who died were excluded from the denominator for the respective time point. To reflect function as close as possible to the time point, we included strength or STS assistance assessments completed within three days of the date of ICU or hospital discharge. To maximize our sample size for 30 s STS, we included the most proximal assessment to each time point. For each measure, we identified paired assessments among participants with completed outcomes at ICU *and* hospital discharge.

We analyzed participant demographics and baseline characteristics for each study independently using descriptive statistics; some data have been previously reported in each study’s main publication [19, 20, 25, 26, 31]. We summarized outcomes using descriptive data. For each measure and time point, we identified the frequency distribution of scores (counts, percentages); identified floor (≥ 15% of participants with minimum score) and ceiling effects (≥ 15% of participants with maximum score) [7]; calculated central tendency [mean (standard deviation) or median (1st, 3rd quartiles) for skewed data]; and assessed normality (Shapiro–Wilk test, *a* = 0.05). For the 30 s STS, we calculated the mean or median time to assessment for each time point, and we considered the “maximum” score based on the upper limit of the 95% confidence interval for sex-matched normative values (29 repetitions for women, 32 for men) [32]. For paired assessments, we calculated each participant’s difference score (hospital minus ICU discharge); the overall group change score [mean (standard deviation) or median (1st, 3rd quartiles) for skewed data]; we compared the difference in assessment scores using a paired t test or Wilcoxon signed-rank test (skewed data), with a two-tailed *a* = 0.05. We also calculated the standard error of the measurement (SEM) and the minimal detectable change at a 90% confidence level (MDC_90_) [20, 33, 34] from paired assessments. We conducted a sensitivity analysis, removing participants who were assigned a score of “0” if they were unable to complete an assessment. Outcome assessment data were analyzed using Stata (v. 15.0, College Station, Texas: StataCorp LP).

We compared 30 s STS scores at each time point against established thresholds for maintenance of physical independence and normative values for community-dwelling older adults [32, 35] matched to our cohort characteristics.

## Results

### Participant demographics

Data from 451 participants enrolled across five studies were analyzed. Participant demographics and baseline characteristics are presented, by study, in Table 1. Most participants were male (*n* = 278, 61.6%) with a mean age between 60 and 66 years. Participants had a median duration of mechanical ventilation between 4 and 8 days, ICU length of stay between 7 and 11 days, hospital length of stay between 22 and 31 days, and mean APACHE II score between 19 and 24. In the next section, we describe results by outcome. Reasons for missing assessments by outcome and time point are in Additional file 1: Figs. 1 and 2.

**Table 1:** Patient demographics and baseline characteristics, by study

	eStimCycle [25]	EXERCISE [26]	I-SURVIVE [20]	TryCYCLE [24]	CYCLE Pilot RCT [19]
Enrolled, N	162	150	40	33	66
Age	61.0 (49.0, 67.0)	60.7 (15.8)	62.0 (17.0)	65.8 (12.2)	61.6 (16.9)
Female, n(%)	55 (44.0)	55 (36.7)	21 (52.5)	16 (48.5)	26 (39.4)
APACHE II	22.3 (7.8)	19.0 (16.0, 23.0)	20.0 (14.0, 28.0)	24.3 (6.7)	23.5 (8.6)
*Admission diagnosis, n (%)*					
Respiratory	65 (40.1)	34 (22.7)	7 (17.5)	19 (57.6)	36 (54.5)
Gastrointestinal	30 (18.5)	0 (0.0)	13 (32.5)	4 (12.1)	8 (12.1)
Cardiovascular	18 (11.1)	23 (15.3)	4 (10.0)	2 (6.1)	3 (4.5)
Sepsis	162 (100.0)^□^	17 (11.3)	6 (15.0)	4 (12.1)	11 (16.7)
Renal	0 (0.0)	7 (4.6)	1 (2.5)	1 (3.0)	2 (3.0)
Non-pulmonary infection	12 (7.4)	0 (0.0)	0 (0.0)	0 (0.0)	0 (0.0)
Cardiac surgery	0 (0.0)	45 (30.0)	0 (0.0)	0 (0.0)	0 (0.0)
Other surgery	0 (0.0)	31 (20.7)	0 (0.0)	2 (6.1)	0 (0.0)
Liver disease/transplant	0 (0.0)	21 (14.0)	0 (0.0)	0 (0.0)	0 (0.0)
Cardiac arrest	0 (0.0)	11 (7.3)	0 (0.0)	0 (0.0)	0 (0.0)
Neurological	0 (0.0)	0 (0.0)	4 (10.0)	0 (0.0)	2 (3.0)
Other	37 (22.8)	11 (7.3)	5 (12.5)	1 (3.0)	4 (6.1)
Charlson Comorbidity Index	2.0 (0.0, 3.0)	*	*	2.2 (2.0)	1.92 (1.6)
Pre-ICU Katz ADL Score	6.0 (6.0, 6.0)	*	6.0 (6.0, 6.0)	5.5 (1.3)	5.65 (0.98)
Duration of MV, days	6.9 (4.0, 10.8)	4.1 (2.1, 7.1)^•^	4.0 (2.0, 9)	8.0 (6.0, 14.0)	8.0 (5.0, 19.0)
ICU length of stay, days	10 (7, 17)	7.0 (6.0, 11.0)	7.0 (4.0, 2.0)	11.0 (7.0, 17.0)	11.0 (8.0, 25.0)
ICU mortality	34 (21.0)	*	†	5 (15.0)	18 (55.0)
Hospital length of stay, days	22.0 (13.0, 39.0)	22.0 (15.0, 36.0)	22.0 (16.0, 48.0)	31.0 (16.0, 42.0)	25.0 (15.0, 45.0)
Hospital mortality	39 (24.0)	*	2 (5.0)	10 (30.0)	22 (67.3)
*Discharge disposition, n(5)*					
Home	74 (60.2)	84 (56.0)	25 (65.8)	13 (56.5)	30 (68.1)
Acute rehabilitation	32 (19.8)	34 (22.7)	5 (13.2)	3 (13.0)	7 (15.9)
Acute hospital	3 (1.9)	8 (5.3)	4 (10.5)	4 (17.4)	5 (11.4)
Chronic care	5 (3.1)	0 (0.0)	2 (5.0)	1 (4.3)	0 (0.0)
Other	48 (29.6)	24 (16.0) ^‡^	0 (0.0)	2 (8.7)	2 (4.5)

Data are presented as Mean (SD) or Median (1st, 3rd quartiles) unless otherwise stated

APACHE II, Acute Physiology and Chronic Health Enquiry II; MV, Mechanical Ventilation

^□^All participants in eStimCycle had sepsis or severe sepsis as this was an inclusion criterion

*Not measured or reported in this study. ^•^ Data available for 137 patients

^†^I-SURVIVE only enrolled patients alive at ICU discharge

^‡^Includes palliative, transitional care or deceased.

### Knee extension

**Fig. 1:**
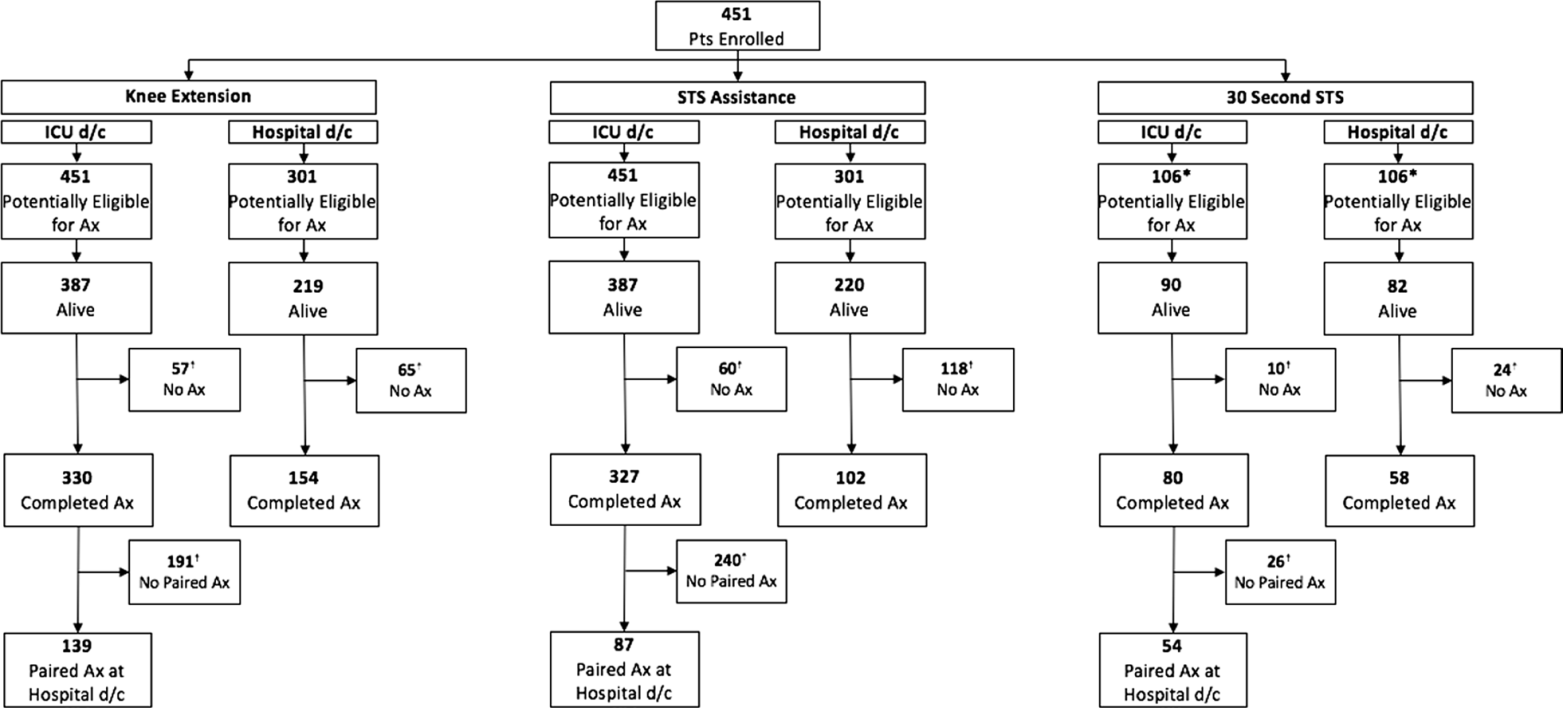
Flowchart of outcome measure assessments for participants enrolled across all five studies. Patients were potentially eligible for an assessment if they were enrolled in an included trial and it was part of the trial protocol to complete an outcome measure assessment at that time point. The number of potentially eligible patients for knee extension and STS assistance is lower at hospital discharge because these outcome measures were not performed at this time point in the EXERCISE trial. Assessments were excluded across all studies if they were performed greater than 72 h from the time of ICU or hospital discharge, respectively. ^☨^Reasons for no assessment are included in Additional file 1: Figs. 1 and 2. *30 Second STS was only assessed in CYCLE Pilot RCT and I-SURVIVE. Pt, Patient; Ax, assessment; STS, sit to stand; KE, knee extension; 30 s STS, 30-second sit to stand; and d/c, Discharge

**Fig. 2:**
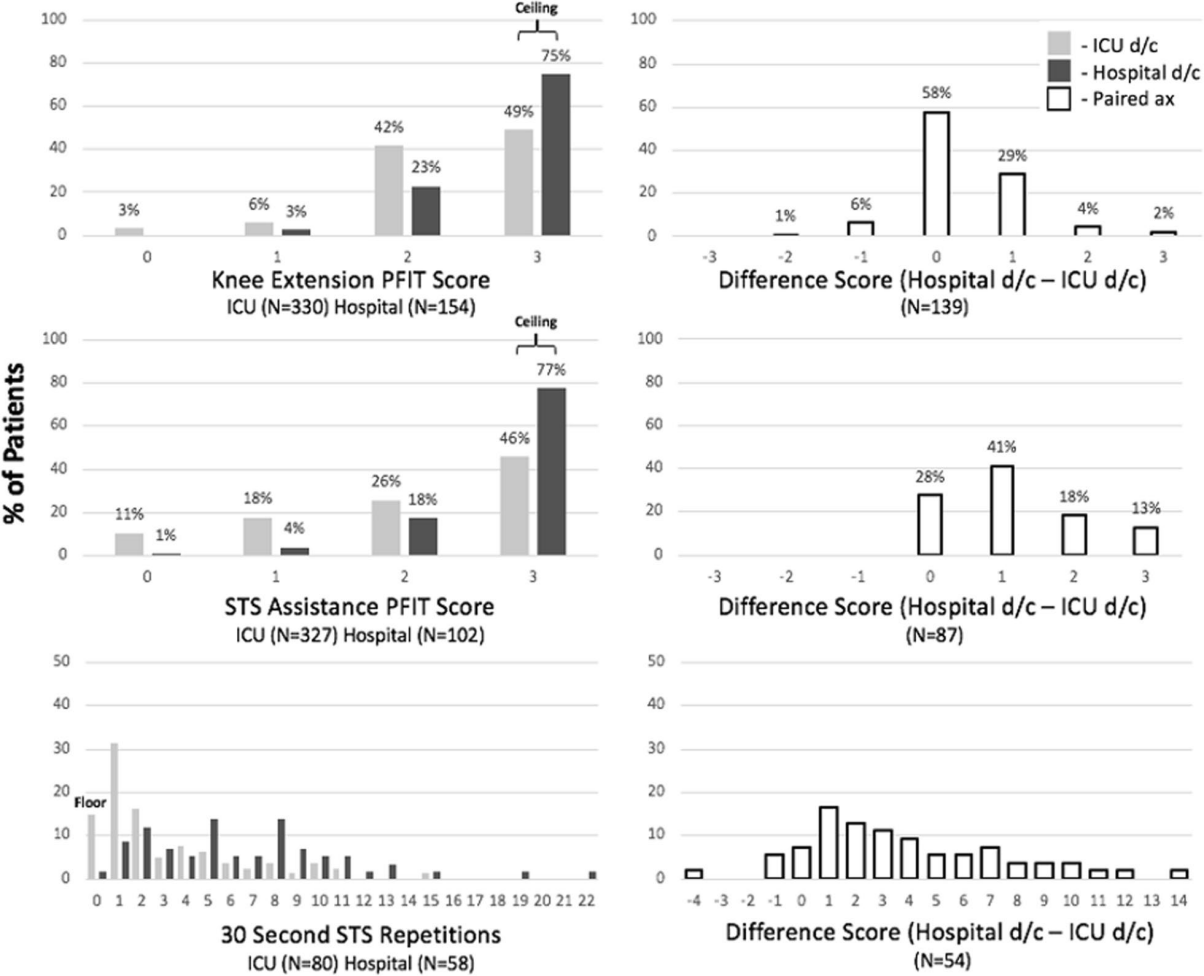
Distribution of scores, including individual assessments (left) and difference scores for paired assessments (right). We considered a floor as ≥ 15% of patients with minimum score and ceiling as ≥ 15% of patients with the maximum score. For 30 s STS, we considered the “maximum” score to be the upper limit of the 95% confidence interval for sex-matched normative values (29 repetitions for women, 32 for men) (Tveter et al., 2014). For patients with assessments completed at ICU and hospital discharge, difference scores were calculated by subtracting scores at ICU discharge from hospital discharge. d/c, Discharge; ax, assessment; and STS, sit to stand

**Fig. 3:**
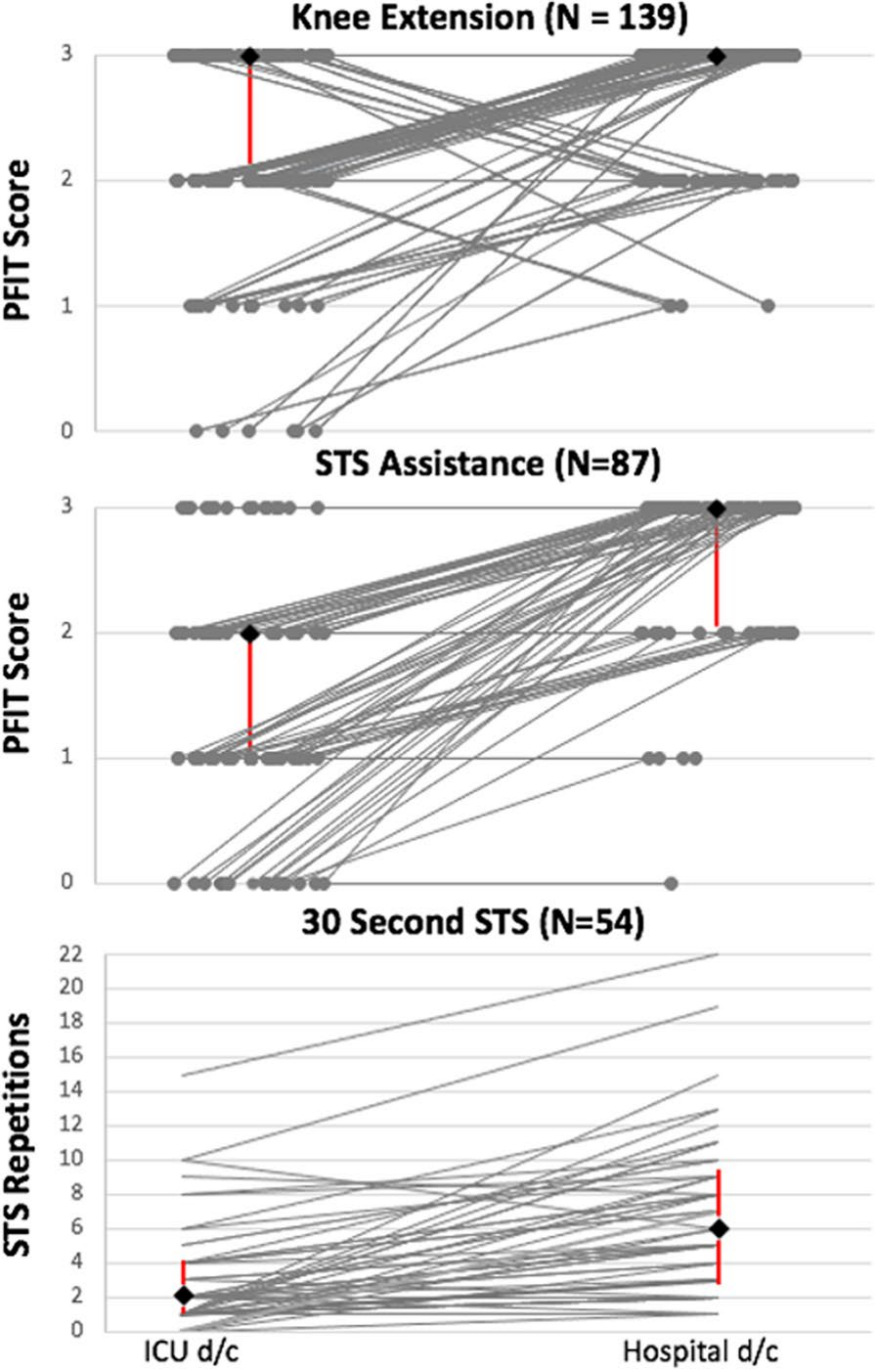
Paired outcome measure scores for participants with assessments at ICU (left) and hospital discharge (right). Each gray line represents one paired assessment. Black diamonds represent median assessment scores at ICU discharge and hospital discharge, for the subset of participants with paired data. Vertical, red bars represent quartiles; bottom bars represent the 1st quartile, and top bars represent the 3rd quartile. For knee extension, where there is no top or bottom bar at hospital discharge, the quartile was the same as the median value. STS, Sit to stand; d/c, discharge

Of 387 potentially eligible participants alive at ICU discharge, 330 (85.3%) had a completed assessment (Fig. 1). The median PFIT-s knee extension score was 2 (2, 3) and a ceiling effect occurred in 48.5% (*n* = 160) (Fig. 2). Of 219 potentially eligible participants alive at hospital discharge, 154 (70.3%) had a completed assessment (Fig. 1). Measurement time points excluded from the parent study protocol accounted for 30 (46.2%) missing assessments (Additional file 1: Fig. 1). The median PFIT-s score was 3 (2, 3) with a ceiling effect in 74.7% (*n* = 115) (Fig. 2). In 139 participants with paired data, the median PFIT-s difference score between ICU and hospital discharge was 0 (0, 1) (Fig. 2; *p* < 0.01) (Fig. 3).

### STS assistance

Of 387 potentially eligible participants alive at ICU discharge, 327 (84.5%) had a completed assessment (Fig. 1). The median STS assistance PFIT-s score was 2 (1, 3) representing assistance with one person, and we calculated a ceiling effect in 45.9% (*n* = 150) (Fig. 2). Of 220 potentially eligible participants alive at hospital discharge, 102 (46.4%) had a completed assessment (Fig. 1). Measurement time points excluded from the parent study protocol accounted for 88 (74.6%) missing assessments (Additional file 1: Fig. 1). The median STS assistance PFIT-s score at hospital discharge was 3 (3, 3) representing no assistance, and a ceiling effect occurred in 77.5% (*n* = 79) (Fig. 2). In 99 participants with paired data, the median difference score between ICU and hospital discharge was 1 (0, 2) (Fig. 2; *p* < 0.01) (Fig. 3).

### 30 s STS

Of 90 potentially eligible participants alive at ICU discharge, 80 (88.9%) had a completed assessment (Fig. 1) with a median 30 s STS score of 2 (1, 5) repetitions, and a floor effect occurred in 15.0% (*n* = 12) (Fig. 2). The median (IQR) time to 30 s STS assessment was 1 day (0, 3) after ICU discharge. Thirty-six participants (45%) used their arms during the test. Of 82 potentially eligible participants alive at hospital discharge, 58 (70.7%) had a completed assessment, with a median 30 s STS score of 6 (3, 9) repetitions (Fig. 1). The median (IQR) time to 30 s STS assessment was 1 day (0, 3) before hospital discharge. Thirty-three participants (57%) used their arms during the test. We did not observe a floor or ceiling effect (Fig. 2). In 54 participants with paired data, the median difference score between ICU and hospital discharge was 3 (1, 6) (*n* = 54; Fig. 2; *p* < 0.01) (Fig. 3). The SEM was 0.51, and the MDC_90_ was 1.19 STS repetition (Additional file 1: Table 3). Sensitivity analyses are included in Additional file 1: Table 4.

**Fig. 4:**
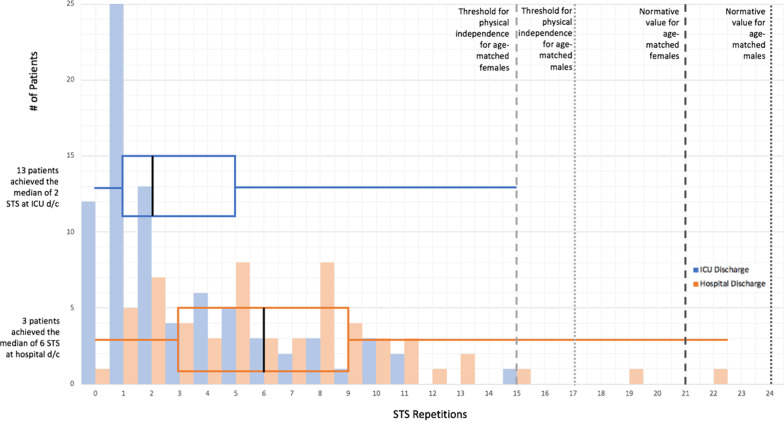
Distribution of 30-Second STS scores at ICU and hospital discharge. We used thresholds to maintain physical independence for moderately active older adults 60–64 years (Rikli and Jones 2013), and normative values for community-dwelling adults aged 60–69 years (Tveter et al. 2014). Blue represents ICU discharge (*n* = 80), and orange represents hospital discharge (*n* = 58). The histogram can be interpreted using the Y-axis. Vertical bars represent the number of patients with each number of STS repetitions. The median scores were 2 (1, 5) STS repetitions at ICU discharge and 6 (3, 9) at hospital discharge. Box plots superimposed upon the histogram represent the median participant score and quartiles. The vertical, black line within the box plot represents the median, while the left side represents the 1st quartile, and the right side represents the 3rd quartile. Tails of the box plot represent the spread of scores, where the left tail represents the minimum, and the right represents the maximum. The horizontal tail lines correspond to the number of patients with the median STS repetitions at each time point

We compared 30 s STS scores for the age range of our cohort against the physical independence thresholds for older adults 60–64 years (females: 15 repetitions, males: 17) and normative values for those 60–69 years (females: 21, males: 24) (Fig. 4). One participant (1.3%) met thresholds for physical independence at ICU discharge, while two participants (3.5%) met thresholds at hospital discharge (Fig. 4). None achieved normative values at ICU discharge, and only one (1.7%) achieved 15 repetitions at hospital discharge (Fig. 4).

## Discussion

Our study represents 451 critically ill participants enrolled across 5 studies, from 3 countries, with synthesized measures at ICU and hospital discharge, and paired assessments between time points. The sample used in this analysis was comparable to previous ICU rehabilitation trial samples with respect to participant characteristics including age [36, 37], sex [36], and clinical characteristics including duration of mechanical ventilation, ICU and hospital length of stay, and APACHE II scores, enhancing generalizability of our findings. The range of APACHE II scores across studies represent moderate-to-severe disease.

Previous research highlighted profound disability experienced by ICU survivors, where only 40% could ambulate at 7 days post-ICU discharge [17]. As a result, outcome measures in this population are plagued by floor and ceiling effects. We identified ceiling effects in knee extension and STS assistance at ICU (~ 50%) and hospital discharge (~ 75%), and floor effects in 30 s STS at ICU discharge (15%). Importantly, we did not observe floor or ceiling effects in the 30 s STS at hospital discharge (Fig. 2). This is in contrast to other measures of physical function for ICU survivors, such as the de Morton Mobility Index and the PFIT-s, which have known limitations at ICU and hospital discharge, respectively. [38] Our data identify the 30 s STS as a promising performance-based functional measure for future ICU longitudinal studies and clinical trials focused on physical function.

ICU survivors demonstrated profound impairments in physical function measured by the 30 s STS at both ICU and hospital discharge. One participant reached normative values and only 2 met or exceeded thresholds required for maintaining physical independence, highlighting the importance of ongoing rehabilitation post-hospital discharge. Small changes in the 30 s STS are likely to be highly relevant to patients’ physical function, providing further justification for the 30 s STS as an outcome measure for clinical trials. [16, 27]

The PFIT-s was developed to measure function at ICU discharge [22], and previous research demonstrates its use at or around ICU discharge [39]; however, a small study documented good reliability and responsiveness post-ICU discharge [20]. Additionally, the potential to use the PFIT-s to prescribe exercise in ICU and at discharge is a unique feature of this test [22]. Data in this current study show that use of individual components of knee extension or STS assistance does not individually demonstrate rigorous outcome metrics for use at ICU or hospital discharge.

To date, many ICU rehabilitation trials are single-centered and enroll small samples [40]. A systematic review and meta-analysis of rehabilitation studies in the ICU summarized 60 RCTs enrolling 5,352 participants [41]. Out of these 60 RCTs, 20 measured muscle strength using the MRC scoring system (16 at ICU, 7 at hospital discharge), 22 reported function (21 at ICU, 15 at hospital discharge; 4 using PFIT-s); 30 s STS outcomes were not reported in this review. The 20 studies measuring muscle strength enrolled 1,713 participants, conducted 1,335 assessments at ICU discharge, and 461 at hospital discharge. The 4 studies reporting the PFIT-s enrolled 316 participants, conducted 167 assessments at ICU, and 53 at hospital discharge. Compared to previous work, our study represents the largest cohort of assessments for PFIT-s components and the 30 s STS at ICU and hospital discharge.

## Implications for future studies

Our observations of the clinimetric properties of the 30 s STS test, including its ease of administration in a clinical or research setting, with no need for expensive equipment, may make it an appropriate and feasible measure of function in future ICU rehabilitation studies. Two approaches to evaluating STS exist: repetition-based (time required to complete a prescribed number of repetitions) [27] or time-based (number of repetitions completed within a prescribed time) [42]. Notably, in a repetition-based approach, participants unable to complete the test cannot be scored (i.e., a floor effect). A time-based approach allows assignment of a score, including a true zero, if a participant is unable to complete the test [27]. In this respect, the 30 s STS is more attractive than outcomes including repetition-based measures, such as the chair stand test in the SPPB, where one component of this battery includes the amount of time required to complete 5 STS repetitions [43]. Thus, for ICU survivors, a time-based approach is more suitable as it allows for a true zero rather than a floor effect, providing a more accurate measure of physical function.

Participants in our sample performed a median of 2 (ICU discharge) and 6 (hospital discharge) sit-to-stand repetitions in 30 s. Community-dwelling patients with stable chronic obstructive pulmonary disease completed an average of 13 sit-to-stand repetitions following pulmonary rehabilitation [44], and those with moderate–severe disease completed 10.8 repetitions [45]. Our data were comparable to the average of 5 repetitions performed by male veterans with an average age of 91 years, using the modified STS (mSTS) [27]. This level of disability lends itself to considering use of a mSTS, which is used with older adults and allows participants to use chair arm rests to perform the test [16]. Our data suggest the ICU survivor population is closer to the geriatric population in physical function at discharge with two potential implications: A mSTS may be best suited and secondly, that MCIDs would be better extrapolated from the geriatric population. While the inability to perform a STS is predictive of falls which is common across all forms of the test [46–48], a difference of 1 mSTS repetition has an odds ratio of 0.75 for decreasing falls risk, and a cutoff of 7 repetitions corresponds to significant decreases in falls risk [27]. The MDC_90_ of the mSTS is 0.7, indicating that a change of 1 or greater is a change beyond that which can be attributed to measurement error [16]. Further, in our cohort of participants, we identified an MDC_90_ of 1.19, representing 1 repetition clinically. Based on these data and our MDC_90_ results, our findings of a median difference score between ICU and hospital discharge of 3 (1, 6) indicate a true change in physical function and is likely functionally meaningful for patients.

Our study has limitations. The 30 s STS test was only performed in the two Canadian studies, and thus, fewer observations may have impacted the precision of our results. Knee extension and STS outcomes were not assessed at hospital discharge in the EXERCISE trial, also limiting our sample size. Our decision to use the most proximal 30 s STS assessment(s) to maximize our sample size may have introduced representation and selection biases in our results. Approximately half of the participants in this study used armrests when completing the 30 s STS test, introducing a variance in the testing protocol. However, we do not believe this contributed to a change in test performance, as participants still demonstrated profound deficits. Our combined data of five international prospective ICU rehabilitation studies also have several strengths, including detailed reasons for missing data, a continuum of measures, measurements at both ICU and hospital discharge, and change in scores between ICU and hospital discharge. We included studies that examined different interventions and somewhat different patient populations with a range of outcome scores. This clinical heterogeneity provides enhanced generalizability of our findings.

## Conclusion

The 30 s STS is relevant to patient function, has good clinimetric and statistical properties, and can be used across the continuum of recovery post-ICU in clinical practice and research. The 30 s STS could be used to assess strength and function at ICU and hospital discharge in moderate to severely ill participants in future studies of physical, nutritional, or metabolic interventions. Until we develop normative values for critically ill patients, our study can inform normative values for ICU survivors and help clinicians contextualize patients’ recovery.

## Supplementary Information

**Additional file 1**. Supplementary Data File: The supplementary data file includes the following: Figure 1. Patient flowchart at ICU discharge, and paired assessments. Figure 2. Patient flowchart at hospital discharge. Table 1. Inclusion/exclusion criteria for each primary study. Table 2. Summary of outcome measures and psychometric properties. Table 3. Descriptive statistics for outcome measures. Table 4. Sensitivity analysis.

## Data Availability

The datasets used during the current study are available from the corresponding author on reasonable request (khome@mcmaster.ca).

## References

[1] Rousseau AF, Prescott HC, Brett SJ (2021). Long-term outcomes after critical illness: recent insights. Crit Care.

[2] Needham DM, Davidson J, Cohen H (2012). Improving long-term outcomes after discharge from intensive care unit: Report from a stakeholders’ conference. Crit Care Med.

[3] Herridge MS, Tansey CM, Matté A (2011). Functional disability 5 years after acute respiratory distress syndrome. N Engl J Med.

[4] Puthucheary ZA, Rawal J, McPhail M (2013). Acute skeletal muscle wasting in critical illness. JAMA.

[5] Waldauf P, Jiroutková K, Krajčová A, Puthucheary Z, Duška F (2020). Effects of rehabilitation interventions on clinical outcomes in critically ill patients: systematic review and meta-analysis of randomized controlled trials. Crit Care Med.

[6] Dinglas VD, Cherukuri SPS, Needham DM (2020). Core outcomes sets for studies evaluating critical illness and patient recovery. Curr Opin Crit Care.

[7] Terwee CB, Bot SDM, de Boer MR (2007). Quality criteria were proposed for measurement properties of health status questionnaires. J Clin Epidemiol.

[8] Parry SM, Nalamalapu SR, Nunna K (2021). Six-minute walk distance after critical illness: a systematic review and meta-analysis. J Intensive Care Med.

[9] Parry SM, Huang M, Needham DM (2017). Evaluating physical functioning in critical care: considerations for clinical practice and research. Crit Care.

[10] Kleyweg RP, Van Der Meché FGA, Schmitz PIM (1991). Interobserver agreement in the assessment of muscle strength and functional abilities in Guillain-Barré syndrome. Muscle Nerve.

[11] Berney S, Skinner EH, Denehy L, Warrillow S (2009). Development of a physical function outcome measure (PFIT) and a pilot exercise training protocol for use in intensive care. Crit Care Resusc.

[12] Jones CJ, Rikli RE, Beam WC (1999). A 30-s chair-stand test as a measure of lower body strength in community-residing older adults. Res Q Exerc Sport.

[13] Ozalevli S, Ozden A, Itil O, Akkoclu A (2007). Comparison of the Sit-to-Stand Test with 6min walk test in patients with chronic obstructive pulmonary disease. Respir Med.

[14] Strassmann A, Steurer-Stey C, Lana KD (2013). Population-based reference values for the 1-min sit-to-stand test. Int J Public Health.

[15] Syddall HE, Martin HJ, Harwood RH, Cooper C, Sayer AA (2009). The SF-36: a simple, effective measure of mobility-disability for epidemiological studies. JNHA J Nutr Health Aging.

[16] McAllister LS, Palombaro KM (2020). Modified 30-second sit-to-stand test: reliability and validity in older adults unable to complete traditional sit-to-stand testing. J Geriatr Phys Ther.

[17] Herridge MS, Chu LM, Matte A (2016). The RECOVER program: disability risk groups and 1-year outcome after 7 or more days of mechanical ventilation. Am J Respir Crit Care Med.

[18] De Jonghe B, Sharshar T, Lefaucheur JP (2002). Paresis acquired in the intensive care unit: a prospective multicenter study. JAMA.

[19] Kho ME, Molloy AJ, Clarke FJ (2019). Multicentre pilot randomised clinical trial of early in-bed cycle ergometry with ventilated patients. BMJ Open Respir Res.

[20] Costigan FA, Rochwerg B, Molloy AJ (2019). I SURVIVE: inter-rater reliability of three physical functional outcome measures in intensive care unit survivors. Can J Anesth Can Anesth.

[21] Fava GA, Tomba E, Sonino N (2012). Clinimetrics: the science of clinical measurements. Int J Clin Pract.

[22] Denehy L, de Morton NA, Skinner EH (2013). A physical function test for use in the intensive care unit: validity, responsiveness, and predictive utility of the physical function ICU test (scored). Phys Ther.

[23] von Elm E, Altman DG, Egger M, Pocock SJ, Gøtzsche PC, Vandenbroucke JP (2007). Strengthening the reporting of observational studies in epidemiology (STROBE) statement: guidelines for reporting observational studies. BMJ.

[24] Kho ME, Molloy AJ, Clarke FJ (2016). TryCYCLE: a prospective study of the safety and feasibility of early in-bed cycling in mechanically ventilated patients. PLoS ONE.

[25] Berney S, Hopkins RO, Rose JW (2021). Functional electrical stimulation in-bed cycle ergometry in mechanically ventilated patients: a multicentre randomised controlled trial. Thorax.

[26] Denehy L, Skinner EH, Edbrooke L (2013). Exercise rehabilitation for patients with critical illness: a randomized controlled trial with 12 months of follow-up. Crit Care.

[27] Applebaum EV, Breton D, Feng ZW (2017). Modified 30-second Sit to Stand test predicts falls in a cohort of institutionalized older veterans. PLoS ONE.

[28] Ciesla N, Dinglas V, Fan E, Kho M, Kuramoto J, Needham D (2011). Manual muscle testing: a method of measuring extremity muscle strength applied to critically ill patients. J Vis Exp JoVE.

[29] Fan E, Ciesla ND, Truong AD, Bhoopathi V, Zeger SL, Needham DM (2010). Inter-rater reliability of manual muscle strength testing in ICU survivors and simulated patients. Intensive Care Med.

[30] Hermans G, Clerckx B, Vanhullebusch T (2012). Interobserver agreement of medical research council sum-score and handgrip strength in the intensive care unit. Muscle Nerve.

[31] TryCYCLE: a prospective study of the safety and feasibility of early in-bed cycling in mechanically ventilated patients. Accessed September 20, 2021. https://journals.plos.org/plosone/article?id=10.1371/journal.pone.0167561. 10.1371/journal.pone.0167561 PMC5193383 28030555

[32] Tveter AT, Dagfinrud H, Moseng T, Holm I (2014). Health-related physical fitness measures: reference values and reference equations for use in clinical practice. Arch Phys Med Rehabil.

[33] Weir JP (2005). Quantifying test-retest reliability using the intraclass correlation coefficient and the SEM. J Strength Cond Res Res J NSCA.

[34] Riddle D, Stratford P. Is This Change Real?: Interpreting Patient Outcomes in Physical Therapy. F.A. Davis; 2013.

[35] Rikli RE, Jones CJ (2013). Development and validation of criterion-referenced clinically relevant fitness standards for maintaining physical independence in later years. Gerontologist.

[36] Moss M, Nordon-Craft A, Malone D (2016). A randomized trial of an intensive physical therapy program for patients with acute respiratory failure. Am J Respir Crit Care Med.

[37] Morris PE, Berry MJ, Files DC (2016). Standardized rehabilitation and hospital length of stay among patients with acute respiratory failure: a randomized clinical trial. JAMA.

[38] Parry SM, Knight LD, Baldwin CE (2020). Evaluating physical functioning in survivors of critical illness: development of a new continuum measure for acute care. Crit Care Med.

[39] Parry SM, Denehy L, Beach LJ, Berney S, Williamson HC, Granger CL (2015). Functional outcomes in ICU—what should we be using? An observational study. Crit Care.

[40] Reid JC, Unger J, McCaskell D, Childerhose L, Zorko DJ, Kho ME (2018). Physical rehabilitation interventions in the intensive care unit: a scoping review of 117 studies. J Intensive Care.

[41] Wang YT, Lang JK, Haines KJ, Skinner EH, Haines TP (2021). Physical rehabilitation in the ICU: a systematic review and meta-analysis. Crit Care Med.

[42] Vaidya T, Chambellan A, de Bisschop C (2017). Sit-to-stand tests for COPD: a literature review. Respir Med.

[43] Guralnik JM, Ferrucci L, Simonsick EM, Salive ME, Wallace RB (1995). Lower-extremity function in persons over the age of 70 years as a predictor of subsequent disability. N Engl J Med.

[44] Zanini A, Aiello M, Cherubino F (2015). The one repetition maximum test and the sit-to-stand test in the assessment of a specific pulmonary rehabilitation program on peripheral muscle strength in COPD patients. Int J Chron Obstruct Pulmon Dis.

[45] Hansen H, Beyer N, Frølich A, Godtfredsen N, Bieler T (2018). Intra- and inter-rater reproducibility of the 6-minute walk test and the 30-second sit-to-stand test in patients with severe and very severe COPD. Int J Chron Obstruct Pulmon Dis.

[46] Buatois S, Miljkovic D, Manckoundia P (2008). Five times sit to stand test is a predictor of recurrent falls in healthy community-living subjects aged 65 and older. J Am Geriatr Soc.

[47] Tiedemann A, Shimada H, Sherrington C, Murray S, Lord S (2008). The comparative ability of eight functional mobility tests for predicting falls in community-dwelling older people. Age Ageing.

[48] Xu W, Chen DW, Jin YB (2015). Incidence and related clinical factors of falls among older Chinese veterans in military communities: a prospective study. J Phys Ther Sci.

