# Supplementary material for: The sit-to-stand test as a patient-centered functional outcome for critical care research: a pooled analysis of five international rehabilitation studies
Source: Crit Care. 2022 Jun 13;26:175. doi: 10.1186/s13054-022-04048-3 (PMC9195216; doi:10.1186/s13054-022-04048-3)
Supplement: Supplementary file 1 — Additional file 1. Supplementary Data File: The supplementary data file includes the following: Figure 1. Patient flowchart at ICU discharge, and paired assessments. Figure 2. Patient flowchart at hospital discharge. Table 1. Inclusion/exclusion criteria for each primary study. Table 2. Summary of outcome measures and psychometric properties. Table 3. Descriptive statistics for outcome measures. Table 4. Sensitivity analysis. [file 13054_2022_4048_MOESM1_ESM.docx]

**Supplementary Data File**

**Table of Contents Page**

Supplementary Figure 1. Patient flowchart at ICU discharge, and paired assessments. 2

Supplementary Figure 2. Patient flowchart at hospital discharge. 3

Supplementary Table 1. Summary of inclusion/exclusion criteria for each primary study. 4

Supplementary Table 2. Summary of outcome measures and psychometric properties. 5

Supplementary Table 3. Descriptive statistics for outcome measures. 6

Supplementary Table 4. Sensitivity analysis. 7

References 8

**Supplementary Figure 1.** Flowchart including reasons for missing assessments at ICU discharge, and paired assessments.


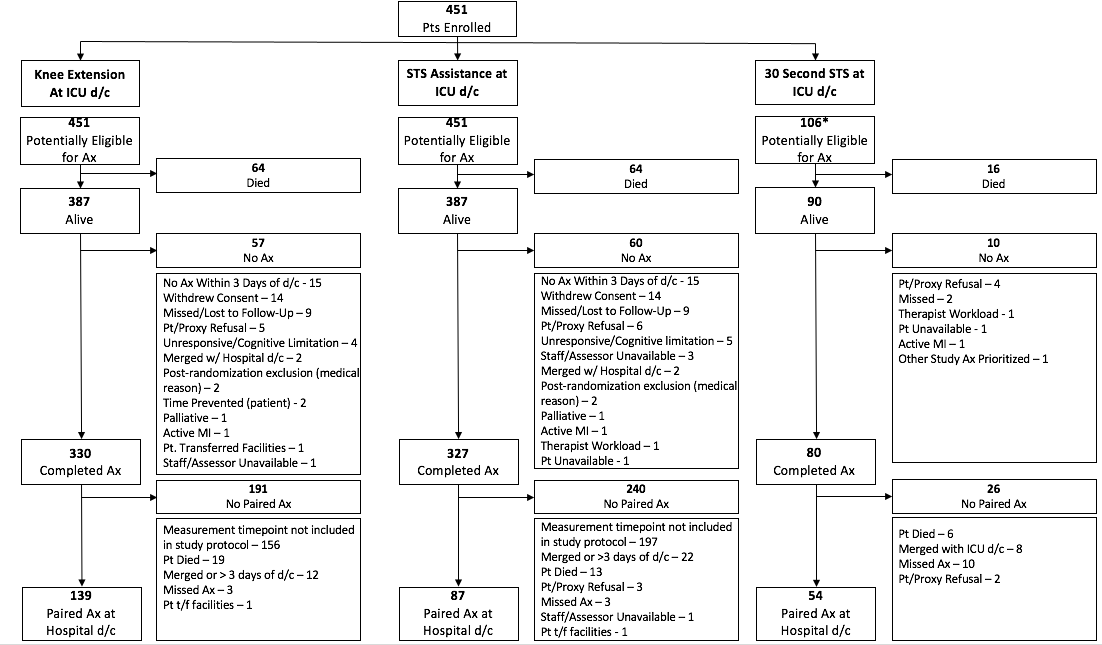


**Legend:** Assessments were excluded across all studies if they were performed greater than 72 hours from the time of ICU or hospital discharge, respectively. *30 Second STS was only assessed in CYCLE Pilot RCT and I-SURVIVE.

**Abbreviations:** Pt – Patient; Ax – Assessment; STS – Sit to Stand; KE – Knee Extension; 30S STS – 30 Second Sit to Stand; d/c – Discharge; w/ - With; MI – Myocardial Infarction.

**Supplementary Figure 2.** Flowchart including reasons for missing outcome measure assessments at hospital discharge.

**
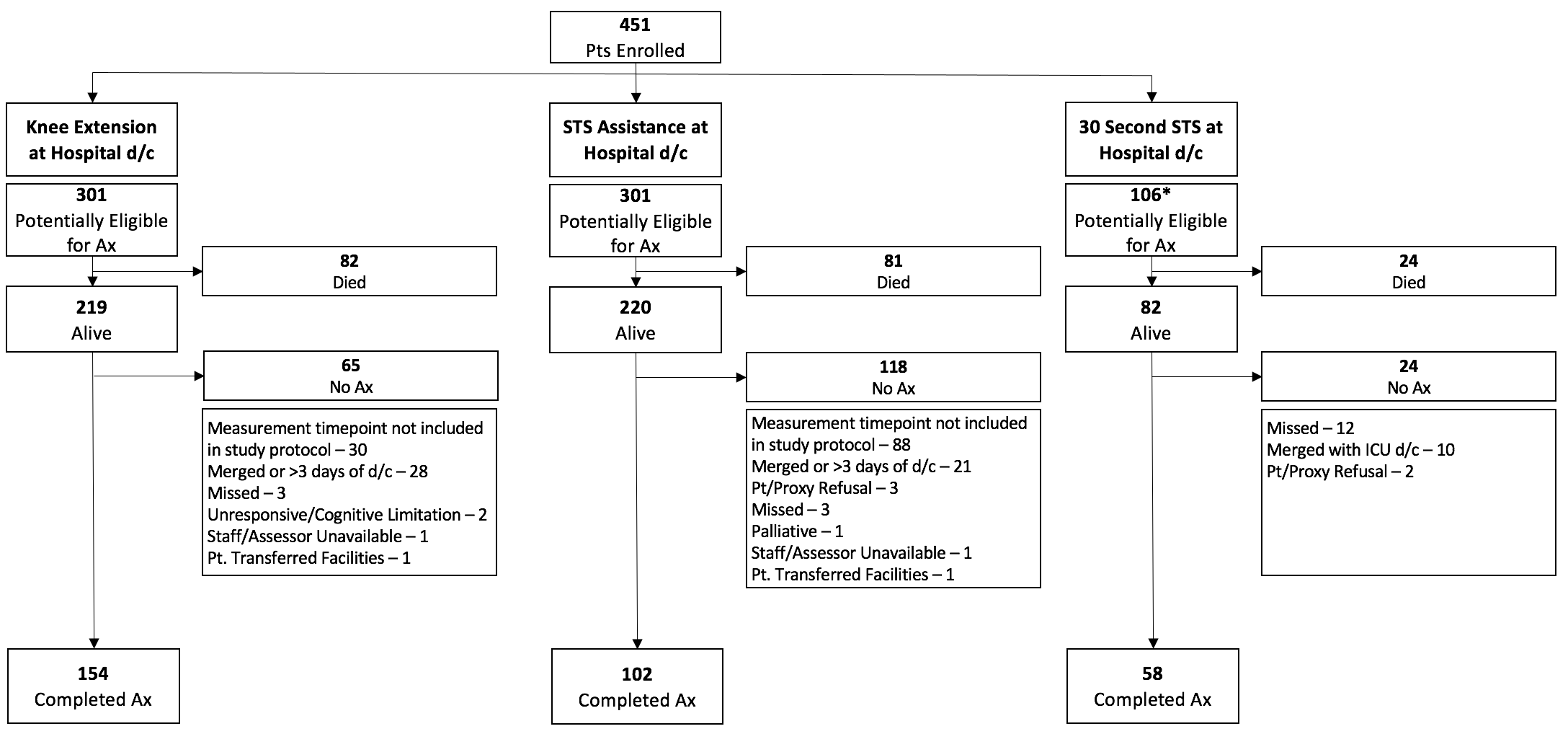
**

**Legend:** Assessments were excluded across all studies if they were performed greater than 72 hours from the time of ICU or hospital discharge, respectively. *30 Second STS was only assessed in CYCLE Pilot RCT and I-SURVIVE.

**Abbreviations:** Pt – Patient; Ax – Assessment; STS – Sit to Stand; KE – Knee Extension; 30S STS – 30 Second Sit to Stand; d/c – Discharge.

**Supplementary Table 1.** Inclusion/exclusion criteria for each primary study.

| **Study** | **Inclusion Criteria** | **Exclusion Criteria** |
| --- | --- | --- |
| I-SURVIVE^1^ | Adult Patients ( ≥18 years old) admitted into the ICU for the first time during the hospital stay were included if they were invasively mechanically ventilated for ≥ 24 hours with a length of stay ≥ 3 days in the ICU; were able to ambulate (with or without gait aid) before hospitalization, discharged from the ICU or ready for discharge, followed by physiotherapy in the ICU; and likely to be available for 2 consecutive week 1 assessments. | Patients were excluded if they were transferred to the study ICU with the length of stay ≥ 7 days, unable to walk ≥ 7 days during hospital stay prior to ICU admission, unable to follow orders in English at baseline, or had acute conditions such as central or peripheral neuromuscular weaknesses. Patients with physical or mental conditions precluding the ability to perform assessments unresolved within 7 days of discharge; or acute myocardial infarction with activity restrictions unresolved within 7 days of discharge were not permitted. Additionally, patients who were pregnant, unlikely to survive, or had palliative goals of care were excluded. |
| TryCYCLE^2^ | Adult patients who were able to ambulate independently (with or without a gait aid) before hospitalization, within the first four days of MV, and the first seven days of ICU admission were included. | Patients were excluded if they were unable to follow orders in English at baseline, could not receive cycling (e.g., did not fit equipment, acute leg fracture), had suspected or confirmed neuromuscular weakness in the legs, were not likely to survive the hospital stay, pregnant, had palliative goals of care, had a temporary pacemaker, or exemptions preventing cycling within the first 4 days of MV. |
| CYCLE Pilot RCT^3^ | Patients who were able to ambulate with or without a gait aid at baseline within the first four days of MV, and the first seven days of ICU admission were included. | Patients were excluded if they were unable to follow orders in English at baseline, unable to receive cycling (e.g., could not fit equipment, acute conditions), had suspected or confirmed neuromuscular weakness, had a temporary pacemaker, had palliative goals of care, were unlikely to survive their hospital stay, or were pregnant. Moreover, patients who had unresolved temporary exemptions within the first 4 days of MV (e.g., hemodynamic, or respiratory instability) were excluded. |
| eStimCycle^4^ | Adult patients (≥18 years of age) who were mechanically ventilated; had sepsis, severe sepsis or systemic inflammatory response syndrome; and expected to need mechanical ventilation ≥48hours and an ICU stay ≥ 4 days post randomisation were included. | Participants were excluded if they did not meet the safety criteria to exercise within 72 hours of meeting the inclusion criteria; or had a neurological diagnosis and were not likely to survive. Enrolled participants were not included for the cognitive outcome assessment at the 6-month follow-up if a score of >3.3 on the Informant Questionnaire on Cognitive Decline in the Elderly Short Form, or a score of >10 on the Alcohol Use Disorders Identification Test before randomisation was reported. |
| The EXERCISE Trial^5^ | Adult participants were included if they were within a 50 km radius of the hospital; had no neurological, spinal or musculoskeletal dysfunction precluding participation in physical rehabilitation; and had stayed in the ICU for at least 5 days. | Patients who were unable to exercise, had permanent neurological damage, had refused consent, had poor English proficiency, or were not likely to survive were excluded. |

**Supplementary Table 2.** Summary of outcome measures and psychometric properties.

| **Outcome** | **Description** | **Interrater Reliability** | **Studies reporting ICU d/c** | **Studies reporting Hospital d/c** |
| --- | --- | --- | --- | --- |
| Knee Extensor Strength^6,7^ | Knee extensor strength was assessed using manual muscle testing (MMT) and scored using the PFIT-s scoring system. PFIT knee extension scored as: 0 – MMT grade 0, 1 or 2; 1 – MMT grade 3; 2 – MMT grade 4; 3 – MMT grade 5. | Good (PFIT-S sum score)  ICC = 0.78 (95% CI = 0.66-0.86)^1^  Population: Adults in ICU | I-SURVIVE  TryCYCLE  CYCLE Pilot RCT  eStimCycle  EXERCISE | I-SURVIVE  TryCYCLE  CYCLE Pilot RCT  eStimCycle |
| STS Assistance^7,8^ | We assessed STS assistance according to the PFIT-S sub-component. A score of 0 represents a patient who is unable to perform the test, 1 represents completing an STS with a two-person assist, 2 represents one-person assist and 3 represents no assist. Higher scores represent better function. | Good (PFIT-S sum score)  ICC = 0.78 (95% CI = 0.66-0.86)^1^  Population: Adults in ICU | I-SURVIVE  TryCYCLE  CYCLE Pilot RCT  eStimCycle  EXERCISE | I-SURVIVE  TryCYCLE  CYCLE Pilot RCT  eStimCycle |
| 30s STS^9–11^ | The 30s STS was assessed by instructing a patient to complete as many STS repetitions as possible in 30 seconds, beginning in a seated position in a chair. Patients were allowed to use arm rests if needed. The number of STS repetitions are counted and represent the patient’s score. Higher repetitions represent greater strength. | Good  ICC = 0.85 (95% CI = 0.76-0.90)^1^  Population: Adults in ICU | I-SURVIVE  CYCLE Pilot RCT | I-SURVIVE  CYCLE Pilot RCT |

**Legend:** ICC values were interpreted as poor (less than 0.5), moderate (between 0.5 and 0.75), good (between 0.75 and 0.9) and excellent (greater than 0.9).^12^

**Abbreviations:** MRC – Medical Research Council; ICC – Intraclass Correlation Coefficient; CI – Confidence Interval; PFIT-S – Physical Function in ICU Test-scored; STS – sit to stand; 30s STS – 30-second sit to stand.

**Supplementary Table 3.** Descriptive statistics for outcome measures, with all studies combined.

|  | **ICU Discharge** | **Hospital Discharge** | **Paired Assessments**  **(ICU and Hospital Discharge)** |
| --- | --- | --- | --- |
| **Knee Extension** | | | |
| Completed Assessments, N | 330 | 154 | 139 |
| PFIT Knee Extension Score, n (%) |  |  |  |
| 0^‡^ | 14 (4.24) | 0 (0.00) | - |
| 1 | 20 (6.06) | 4 (2.60) | - |
| 2 | 136 (41.21) | 35 (22.73) | - |
| 3^†^ | 160 (48.48) | 115 (74.68) | - |
| Mean (SD) | 2.33 (0.78) | 2.72 (0.51) | 0.36 (0.79) |
| SEM |  |  | 0.067 |
| MDC_90_ |  |  | 0.16 |
| Median (1^st^, 3^rd^ Quartiles) | 2 (2, 3) | 3 (2, 3) | 0 (0, 1)* |
| Shapiro Wilk W (p-value) | 0.95 (<0.01) | 0.89 (<0.01) | 0.98 (0.01) |
| Wilcoxon Signed-Rank Test, z (p-value) | - | - | 5.10 (<0.01) |
| **Sit to Stand Assistance** | | | |
| Completed Assessments, N | 327 | 102 | 87 |
| PFIT Sit to Stand Assistance Score, n (%) |  |  |  |
| 0^‡^ | 35 (10.70) | 1 (0.98) | - |
| 1 | 58 (17.74) | 4 (3.92) | - |
| 2 | 84 (25.69) | 18 (17.65) | - |
| 3^†^ | 150 (45.87) | 79 (77.45) | - |
| Mean (SD) | 2.07 (1.03) | 2.72 (0.59) | 1.16 (0.98) |
| SEM |  |  | 0.11 |
| MDC_90_ |  |  | 0.24 |
| Median (1^st^, 3^rd^ Quartiles) | 2 (1, 3) | 3 (3, 3) | 1 (0, 2)* |
| Shapiro Wilk W (p-value) | 0.99 (<0.01) | 0.75 (<0.01) | 0.98 (0.24) |
| Wilcoxon Signed-Rank Test, z (p-value) | - | - | 7.63 (<0.01) |
| **30 Second Sit to Stand** | | | |
| Completed Assessments, N | 80 | 58 | 54 |
| Mean (SD) | 3.05 (3.25) | 6.53 (4.48) | 3.81 (3.72) |
| SEM | - | - | 0.51 |
| MDC_90_ | - | - | 1.19 |
| Median (1^st^, 3^rd^ Quartiles) | 2 (1, 5) | 6 (3, 9) | 3 (1, 6)* |
| 30 Second Sit to Stand Repetitions, n (%) |  |  |  |
| 0^‡^ | 12 (15.00) | 1 (1.72) | - |
| 1-5 | 53 (66.25) | 27 (46.55) | - |
| 6-10 | 12 (15.00) | 21 (36.2) | - |
| 11-15 | 1 (1.25) | 7 (12.07) | - |
| 16-20 | 1 (1.25) | 1 (1.72) | - |
| 21-25 | 0 (0.0) | 1 (1.72) | - |
| Range | 0 to 15 | 0 to 22 | -4 to 14* |
| Shapiro Wilk W (p-value) | 0.84 (<0.01) | 0.93 (0.02) | 0.96 (0.069) |
| Wilcoxon Signed-Rank Test, z (p-value) | - | - | 5.81 (<0.01) |

**Legend:** PFIT knee extension scored as: 0 – manual muscle testing (MMT) grade 0, 1 or 2; 1 – MMT grade 3; 2 – MMT grade 4; 3 – MMT grade 5. PFIT sit to stand assistance scored as: 0 – unable to perform; 1 – assist x2; 2 – assist x1; 3 – no assist. (Denehy et al., 2013).

^‡^ Floor. ^†^ Ceiling. * Data represent difference scores, calculated by subtracting ICU discharge from hospital discharge scores. – represents a statistic that is not applicable for a given data set.

**Supplementary Table 4.** Descriptive statistics for outcome measures, excluding particpants who did not complete assessments and were scored as “0” (unable to perform) according to the PFIT-s.

|  | **ICU Discharge** | **Hospital Discharge** | **Paired Assessments** |
| --- | --- | --- | --- |
| **Knee Extension** | | | |
| Completed Assessments, N | 327 | 154 | 139 |
| PFIT Knee Extension Score, n (%) |  |  | - |
| 0^‡^ | 11 (3.36) | 0 (0.00) | - |
| 1 | 20 (6.12) | 4 (2.60) |  |
| 2 | 136 (41.59) | 35 (22.73) | - |
| 3^†^ | 160 (48.93) | 115 (74.68) | - |
| Mean (SD) | 2.36 (0.75) | 2.72 (0.51) | 0.36 (0.79) |
| Median (1^st^, 3^rd^ Quartiles) | 2 (2, 3) | 3 (2, 3) | 0 (0, 1)* |
| Shapiro Wilk W (p-value) | 0.95 (<0.01) | 0.89 (<0.01) | 0.98 (0.01) |
| Wilcoxon Signed-Rank Test, z (p-value) | - | - | 5.10 (<0.01) |
| **Sit to Stand Assistance** | | | |
| Completed Assessments, N | 309 | 101 | 78 |
| PFIT Sit to Stand Assistance Score, n (%) |  |  |  |
| 0^‡^ | 17 (5.50) | 0 (0.00) | - |
| 1 | 58 (18.77) | 4 (3.96) | - |
| 2 | 84 (27.18) | 18 (17.82) | - |
| 3^†^ | 150 (48.54) | 79 (78.22) | - |
| Mean (SD) | 2.19 (0.93) | 2.74 (0.52) | 1.03 (0.87) |
| Median (1^st^, 3^rd^ Quartiles) | 2 (2, 3) | 3 (3, 3) | 1 (0, 2)* |
| Shapiro Wilk W (p-value) | 0.98 (<0.01) | 0.83 (<0.01) | 0.97 (0.06) |
| Wilcoxon Signed-Rank Test, z (p-value) | - | - | 7.17 (<0.01) |
| **30 Second Sit to Stand** | | | |
| Completed Assessments, N | 68 | 58 | 48 |
| Mean (SD) | 3.59 (3.24) | 6.53 (4.48) | 3.56 (3.76) |
| Median (1^st^, 3^rd^ Quartiles) | 2 (1, 5) | 6 (3, 9) | 3 (1, 6)* |
| 30 Second Sit to Stand Repetitions, n (%) |  |  |  |
| 0^‡^ | 0 (0.00) | 1 (1.72) | - |
| 1-5 | 53 (77.94) | 27 (46.55) | - |
| 6-10 | 12 (17.64) | 21 (36.2) | - |
| 11-15 | 1 (1.47) | 7 (12.07) | - |
| 16-20 | 1 (1.47) | 1 (1.72) | - |
| 21-25 | 0 (0.00) | 1 (1.72) | - |
| Range | 1 to 15 | 0 to 22 | -4 to 14* |
| Shapiro Wilk W (p-value) | 0.85 (<0.01) | 0.93 (0.02) | 0.94 (0.02) |
| Wilcoxon Signed-Rank Test, z (p-value) | - | - | 5.37 (<0.01) |

**Legend:** PFIT knee extension scored as: 0 – manual muscle testing (MMT) grade 0, 1 or 2; 1 – MMT grade 3; 2 – MMT grade 4; 3 – MMT grade 5. PFIT sit to stand assistance scored as: 0 – unable to perform; 1 – assist x2; 2 – assist x1; 3 – no assist. (Denehy et al., 2013).

^‡^ Floor. ^†^ Ceiling. * Data represent difference scores, calculated by subtracting ICU discharge from hospital discharge scores. – represents a statistic that is not applicable for a given data set.

**References**

1. Costigan FA, Rochwerg B, Molloy AJ, et al. I SURVIVE: inter-rater reliability of three physical functional outcome measures in intensive care unit survivors. *Can J Anesth Can Anesth*. 2019;66(10):1173-1183. doi:10.1007/s12630-019-01411-x

2. Kho ME, Molloy AJ, Clarke FJ, et al. TryCYCLE: A Prospective Study of the Safety and Feasibility of Early In-Bed Cycling in Mechanically Ventilated Patients. *PLOS ONE*. 2016;11(12):e0167561. doi:10.1371/journal.pone.0167561

3. Kho ME, Molloy AJ, Clarke FJ, et al. Multicentre pilot randomised clinical trial of early in-bed cycle ergometry with ventilated patients. *BMJ Open Respir Res*. 2019;6(1):e000383. doi:10.1136/bmjresp-2018-000383

4. Berney S, Hopkins RO, Rose JW, et al. Functional electrical stimulation in-bed cycle ergometry in mechanically ventilated patients: a multicentre randomised controlled trial. *Thorax*. 2021;76(7):656-663. doi:10.1136/thoraxjnl-2020-215093

5. Denehy L, Skinner EH, Edbrooke L, et al. Exercise rehabilitation for patients with critical illness: a randomized controlled trial with 12 months of follow-up. *Crit Care*. 2013;17(4):R156. doi:10.1186/cc12835

6. Kleyweg RP, Van Der Meché FGA, Schmitz PIM. Interobserver agreement in the assessment of muscle strength and functional abilities in Guillain-Barré syndrome. *Muscle Nerve*. 1991;14(11):1103-1109. doi:10.1002/mus.880141111

7. Denehy L, de Morton NA, Skinner EH, et al. A Physical Function Test for Use in the Intensive Care Unit: Validity, Responsiveness, and Predictive Utility of the Physical Function ICU Test (Scored). *Phys Ther*. 2013;93(12):1636-1645. doi:10.2522/ptj.20120310

8. Berney S, Skinner EH, Denehy L, Warrillow S. Development of a physical function outcome measure (PFIT) and a pilot exercise training protocol for use in intensive care. *Crit Care Resusc*. Published online June 2009. Accessed November 19, 2021. https://search.informit.org/doi/abs/10.3316/informit.513699052421471

9. Ciesla N, Dinglas V, Fan E, Kho M, Kuramoto J, Needham D. Manual Muscle Testing: A Method of Measuring Extremity Muscle Strength Applied to Critically Ill Patients. *J Vis Exp JoVE*. 2011;(50):2632. doi:10.3791/2632

10. Fan E, Ciesla ND, Truong AD, Bhoopathi V, Zeger SL, Needham DM. Inter-rater reliability of manual muscle strength testing in ICU survivors and simulated patients. *Intensive Care Med*. 2010;36(6):1038-1043. doi:10.1007/s00134-010-1796-6

11. Hermans G, Clerckx B, Vanhullebusch T, et al. Interobserver agreement of medical research council sum-score and handgrip strength in the intensive care unit. *Muscle Nerve*. 2012;45(1):18-25. doi:10.1002/mus.22219

12. Koo TK, Li MY. A Guideline of Selecting and Reporting Intraclass Correlation Coefficients for Reliability Research. *J Chiropr Med*. 2016;15(2):155-163. doi:10.1016/j.jcm.2016.02.012
